# The Gut Dysmotility Questionnaire for Parkinson's disease: Insights into development and pretest studies

**DOI:** 10.3389/fneur.2023.1149604

**Published:** 2023-03-28

**Authors:** Vanessa Raeder, Lucia Batzu, Robert Untucht, Annekathrin Fehre, Alexandra Rizos, Valentina Leta, Renate Schmelz, Jochen Hampe, Sevasti Bostantjopoulou, Zoe Katsarou, Alexander Storch, Heinz Reichmann, Björn Falkenburger, K. Ray Chaudhuri, Lisa Klingelhoefer

**Affiliations:** ^1^Department of Neurology, University Hospital Dresden, Technische Universität Dresden, Dresden, Germany; ^2^Parkinson's Foundation Centre of Excellence, King's College Hospital, London, United Kingdom; ^3^Department of Basic and Clinical Neuroscience, King's College London, Institute of Psychiatry, Psychology, and Neuroscience, The Maurice Wohl Clinical Neuroscience Institute, London, United Kingdom; ^4^Department of Neurology and Experimental Neurology, Charité-Universitätsmedizin Berlin, Corporate Member of Freie Universität Berlin and Humboldt Universität zu, Berlin, Germany; ^5^Department of Neurology, Elblandklinikum Meißen, Meißen, Germany; ^6^Department of Internal Medicine I, University Hospital Dresden, Technische Universität Dresden, Dresden, Germany; ^7^3rd Department of Neurology, Aristotle University of Thessaloniki, Thessaloniki, Greece; ^8^Department of Neurology, University of Rostock, Rostock, Germany; ^9^German Center for Neurodegenerative Diseases (DZNE) Rostock/Greifswald, Rostock, Germany

**Keywords:** bowel movement, constipation, gut, questionnaire, Parkinson's disease, cognitive pretest, non-motor symptoms

## Abstract

**Objective:**

A total of 48% of patients with Parkinson's disease (PD) present symptoms of gastrointestinal dysfunction, particularly constipation. Furthermore, gastrointestinal tract (GIT)-related non-motor symptoms (NMSs) appear at all stages of PD, can be prodromal by many years and have a relevant impact on the quality of life. There is a lack of GIT-focused validated tools specific to PD to assess their occurrence, progress, and response to treatment. The aim of this study was to develop and evaluate a novel, disease- and symptom-specific, self-completed questionnaire, titled Gut Dysmotility Questionnaire (GDQ), for screening and monitoring gastrointestinal dysmotility of the lower GIT in patients with PD.

**Methods:**

In phase 1, a systematic literature review and multidisciplinary expert discussions were conducted. In phase 2, cognitive pretest studies comprising standard pretests, interviews, and evaluation questionnaires were performed in patients with PD (*n* = 21), age- and sex-matched healthy controls (HC) (*n* = 30), and neurologists (*n* = 11). Incorporating these results, a second round of cognitive pretests was performed investigating further patients with PD (*n* = 10), age- and sex-matched HC (*n* = 10), and neurologists (*n* = 5). The questionnaire was adapted resulting in the final GDQ, which underwent cross-cultural adaptation to the English language.

**Results:**

We report significantly higher GDQ total scores and higher scores in five out of eight domains indicating a higher prevalence of gastrointestinal dysmotility in patients with PD than in HC (*p* < 0.05). Cognitive pretesting improved the preliminary GDQ so that the final GDQ was rated as relevant (100/100%), comprehensive (100/90%), easy to understand concerning questions and answer options (100/90%), and of appropriate length (80/100%) by neurologists and patients with PD, respectively. The GDQ demonstrated excellent internal consistency (Cronbach‘s alpha value of 0.94). Evidence for good construct validity is given by moderate to high correlations of the GDQ total score and its domains by intercorrelations (*r*_*s*_ = 0.67–0.91; *p* < 0.001) and with validated general NMS measures as well as with specific items that assess gastrointestinal symptoms.

**Interpretation:**

The GDQ is a novel, easy, and quick 18-item self-assessment questionnaire to screen for and monitor gastrointestinal dysmotility with a focus on constipation in patients with PD. It has shown high acceptance and efficacy as well as good construct validity in cognitive pretests.

## 1. Introduction

Patients with Parkinson's disease (PwPD) present with motor and non-motor symptoms (NMSs). Although the clinical diagnosis of Parkinson's disease (PD) is primarily based on motor symptoms caused by dopamine deficiency (1–3), NMSs are increasingly relevant diagnostic criteria for PD (2, 4).

A broad spectrum of NMSs is already prevalent in the prodromal stage, several years before motor symptoms appear. NMSs are common in all PwPD and occur at all stages of the disease (1, 4–6). Several studies have shown that NMSs have a greater impact on health-related quality of life (HRQoL) in PwPD in comparison to motor symptoms (7). Therefore, evaluation, monitoring, and treatment of NMSs are crucial for a holistic approach to PwPD.

In particular, gastrointestinal dysfunctions are common, prominent, and troublesome NMSs, which can impair the absorption of oral anti-PD drugs and potentially affect HRQoL in PwPD (5, 8–13). Up to 48% of PwPD present gastrointestinal symptoms, particularly constipation (14). There are global NMS tools such as the Non-Motor Symptoms Questionnaire (NMSQuest) (12) and the NMSS (15) that ask about gastrointestinal symptoms next to other NMS but more in a sense if there is an involvement of the gastrointestinal tract (GIT) or not. Specific questionnaires such as the SCOPA-AUT (16) assess the whole GIT and autonomic symptoms, but there is still a lack of validated disease- and symptom-specific instruments to screen for and monitor gastrointestinal dysmotility of the lower GIT with a focus on constipation in PD nor are there validated instruments for other diseases that could be transferred and used in PwPD. This is an unmet need based on the following rationale: Constipation is an important symptom in the prodromal stage of PD and is associated with a higher risk of PD development (6, 8, 17, 18). Furthermore, in the majority of patients with PD, it is hypothesized that the pathophysiological process leading to clinically manifested PD starts in the gut (19–24). Indeed, pathological alpha-synuclein deposits could already be detected in the entire gastrointestinal tract 20 years before diagnosis (20, 21, 25).

Thus, there is a need for a questionnaire that can detect gut dysmotility, and the questionnaire should be applicable to screen people who are at risk of PD development. Furthermore, constipation is evident throughout the whole course of PD (15, 26), so that the assessment and monitoring of gastrointestinal motility and constipation are necessary for any patient with PD on a regular basis. In addition, treatment effects should be recognized when monitoring these symptoms as well as their effect on HRQoL. The need for such a questionnaire has already been expressed by the Movement Disorders Society (MDS) (27). In addition, the development of scales and questionnaires such as the NMSQuest (12) or the symptom-specific Parkinson's Disease Sleep Scale (28) has resulted in a better understanding of NMS and enhanced the diagnostic and treatment approaches in PD.

Therefore, we developed the Gut Dysmotility Questionnaire (GDQ) as a screening and monitoring tool for gastrointestinal dysmotility with a focus on constipation in international collaboration (29). A comprehensive cognitive pretest study was performed including PwPD, and healthy controls (HC) as well as neurologists. This resulted in the final GDQ as a disease- and symptom-specific, self-completed, short, and holistic questionnaire to screen for and monitor gastrointestinal dysmotility of the lower GIT in PwPD.

## 2. Materials and methods

### 2.1. Phase 1: Development of the preliminary GDQ

In phase 1, a systematic literature search was performed to identify questionnaires and to reveal relevant questions in relation to lower gastrointestinal tract symptoms. In the PubMed search, we used combinations of the key terms “Constipation AND Parkinson,” “Bowel Movement AND Parkinson,” and “Constipation AND Questionnaire,” including all articles in English and German of any type up to October 2018. A selection of questions in English was developed and discussed in repetitive multidisciplinary expert group meetings. Hereby, the preliminary GDQ (pGDQ) was developed.

### 2.2. Phase 2: Standard and cognitive pretest study of the GDQ

The objective of this study was to perform standard and cognitive pretests on PwPD, HC, and neurologists using the German version of the pGDQ to verify its wording and effectiveness (30) as well as to further refine the questionnaire. Phase 2a covered the first standard and cognitive pretest. Hereafter, the GDQ was adapted and pretested again in phase 2b.

#### 2.2.1. Study design and procedures

The standard and cognitive pretest study was performed as an open, prospective, single-center evaluation study at the Department of Neurology of the Technische Universität Dresden (TUD), Germany.

The cognitive pretests included structured interviews and evaluation questionnaires in the following three groups: patients with idiopathic PD, age and sex-matched HC, and neurologists specialized in movement disorders.

Patients with Parkinson's disease were consecutively recruited in the movement disorders-specialized out- and in-patient clinics of the Department of Neurology of TUD. The HC were mainly relatives and companions of the investigated PwPD. Ethical approval (EK 518122019) was granted by the ethics committee of TUD. All participants gave written informed consent before any study-related procedure was initiated.

In phase 2a, a standardized study protocol was performed in PwPD and HC with a collection of sociodemographic and disease-related data. In addition, validated PD-specific scales and questionnaires were used to obtain a clinical impression of motor and non-motor burden (Montreal Cognitive Assessment, Hoehn & Yahr stage, clinical impression of severity index for PD, Beck Depression Inventory), general medical health state (clinical global impression, patient global impression), and HRQoL (Parkinson's Disease Quality of Life Questionnaire eight, EQ-5D-5L). Furthermore, questionnaires assessing gastrointestinal symptoms (MDS-UPDRS part I question 1.11, SCOPA-AUT, NMSQuest), influencing factors, and habits such as smoking and caffeine consumption, and physical activity were recorded. The standard and cognitive pretests were interview-based on a specifically prepared interview guideline and protocol and conducted with all PwPD and HC (30). The PwPD and HC completed the pGDQ as well as the evaluation questionnaire themselves. While doing so, verbal and non-verbal reactions were observed by the study personnel. Following completion, each individual question of the pGDQ as well as any unusual verbal and non-verbal reactions observed during the completion of the pGDQ were discussed in a personal interview with the participants. Techniques of think-aloud, verbal probing, and a confidence rating were used (30). For the think-aloud method, the participant was asked to express his or her thoughts on each question before and during answering the question. Patients were encouraged to reflect on all possible thoughts on each question. In verbal probing, specific questions were asked about the answer types of the questionnaire. For confidence rating, participants were asked to indicate how correctly they answered each question. If uncertainties were stated, the participants were asked why they felt so. In addition, each data point of the interview protocols was quantitatively and qualitatively analyzed by the developers for further guidance. The participants themselves were also encouraged to make valuable and well-structured suggestions for the improvement of the pGDQ. The time taken to complete the questionnaire was recorded.

The study protocol for the neurologists was more concise and required demographic data and a level of expertise in the field of neurology. Each neurologist scored a total of four pGDQ questionnaires (two completed by PwPD and two by HC) using a provided scoring guide and further completed an evaluation questionnaire for cognitive pretesting.

The evaluation questionnaire of the pGDQ was the same in all three study groups. It contained simple yes and no answers with an additional free text option for remarks and was adopted from the literature (31). In addition, the neurologists evaluated the different domains and the scoring system of the pGDQ.

The pGDQ, the scoring guide, and the evaluation questionnaire were adapted to the results of phase 2a resulting in the prefinal GDQ (pfGDQ) which was retested in phase 2b investigating further PwPD and HC as well as neurologists who had already participated in phase 2a. The standardized study protocol of phase 2a was shortened and performed with the standard and cognitive pretests in all PwPD and HC. The PwPD and HC completed the pGDQ as well as the evaluation questionnaire themselves, followed by an interview as in phase 2a.

The study protocol for the neurologists was repeated, and each neurologist scored a total of four pGDQ questionnaires (two completed by PwPD and two by HC) using a provided scoring guide and completed an evaluation questionnaire for cognitive pretesting.

#### 2.2.2. Inclusion and exclusion criteria

Healthcare professionals were included if certified as neurologists and study nurses, each with specific knowledge in movement disorders or geriatrics. Participants of the PD study group had to be diagnosed with idiopathic PD based on the clinical diagnostic criteria (2) and had to be at least 18 years old. HC had to be between 30 and 80 years old.

The exclusion criteria for the PD study group were any diagnosis of atypical or secondary PD, severe memory impairment, or any uncontrolled psychiatric illness such as psychosis. HC was excluded if they were diagnosed with severe memory impairment or any acute and uncontrolled neurological, psychiatric, or gastrointestinal concomitant diseases (e.g., psychosis and gastrointestinal infection).

#### 2.2.3. Data analysis

Data analysis was performed using SPSS. Demographic and clinical characteristics of phase 2a and phase 2b were analyzed using non-parametric tests as the data were mostly not normally distributed.

For the evaluation of the preliminary and prefinal GDQ, the following parameters were analyzed: data quality (< 10% missing data and more than 90% calculable scores), floor and ceiling effects below 15%, and skewness between −1 and +1. The reliability of both questionnaires was explored with Cronbach's alpha (>0.70), inter-item correlation (0.20–0.75), item homogeneity coefficient (>0.15), and corrected item-total correlation (≥0.30). Spearman's rank correlation coefficients were considered “weak” if the r_s_-value was < 0.3, “moderate” if 0.3–0.59, and “high” if >0.60 (32, 33).

Data from the standard pretests, cognitive pretests, and evaluation questionnaires were analyzed with qualitative and quantitative methods, including descriptive tests. The collected data were categorized and quantified using an adapted Classification Coding Scheme (CCS) (34). A *P*-value of < 0.05 was considered to be statistically significant.

### 2.3. Cross-cultural adaptation of the GDQ

The cross-cultural adaptation of the GDQ followed international guidelines with translation from German to English language and vice versa (35). Detailed information will be published in another scientific article.

## 3. Results

### 3.1. Phase 1

**Figure 1:**
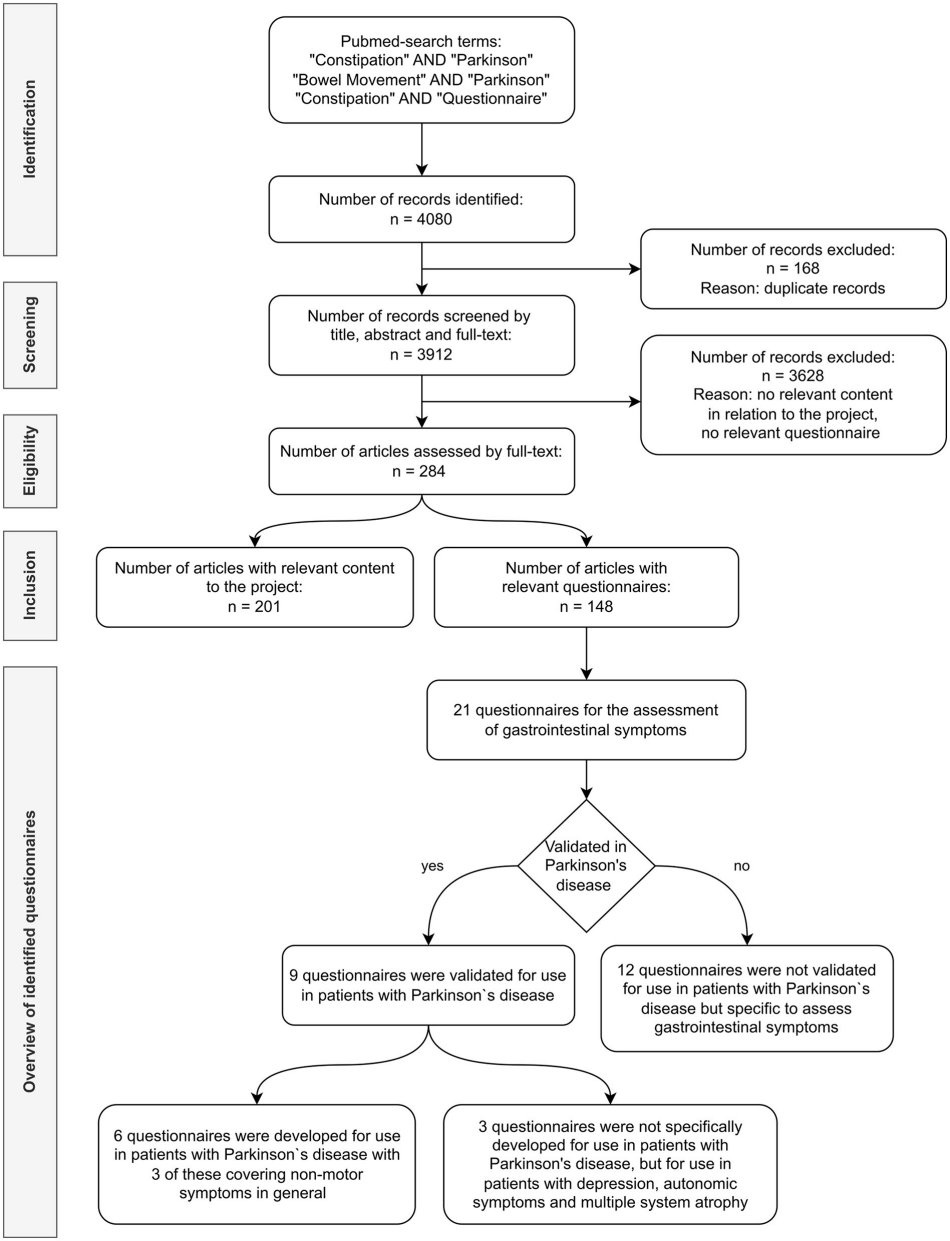
Flowchart summarizing the literature search.

Based on a systematic literature search (Figure 1) and identified questionnaires, a selection of questions in English was developed aiming to cover all relevant domains in relation to gastrointestinal dysmotility and PD. In repetitive multidisciplinary expert group meetings including internationally recognized movement disorders specialists (*N* = 12), gastrointestinal specialists (*N* = 2), and PD specialist nurses and study nurses (*N* = 2), the following points were discussed: relevant questions/content, design of questions and answer possibilities, meaningful domains to merge questions, the relevance of influencing factors and associated symptoms, and scoring system.

Phase 1 resulted in the pGDQ, which consisted of 16 questions with eight sub-questions, comprising 24 questions in total. The questions were assigned to eight different domains: frequency, duration, severity, consistency, assistance, pain, quality of life, and development (**Table 2**). Answers were mainly provided by a four-item unipolar response scale. In the domain of stool consistency, answers were assessed in a table with small drawings for visualization. The answer options in the domain development were designed as a visual analog scale, ranging from constipation “improving” over “stable” to “worsening.” As a scoring method, a basic summation of all answers was chosen so that the total score of the pGDQ could range from 0 to 74 points with higher values implicating worse gastrointestinal dysmotility.

### 3.2. Phase 2

#### 3.2.1. Phase 2a study: Cognitive pretests of the preliminary GDQ

##### 3.2.1.1. Characteristics of the study sample

In phase 2a, 21 PwPD and 30 HC as well as 11 neurologists were included. Demographic, motor, and non-motor characteristics of PwPD and HC are summarized in Table 1.

**Table 1:** Demographic, motor, and non-motor characteristics of patients with Parkinson's disease (PD) and healthy controls of the phase 2a study.

	**PD patients (*n =* 21)**	**Healthy controls (*n =* 30)**	***P*-value**
Age (years) (minimum-maximum)	65.52 ± 8.63 (49.00–80.00)	59.70 ± 14.02 (30.00–80.00)	0.168
Sex (m/f)	12/9	16/14	0.788
Education (years) (minimum-maximum)	10.81 ± 1.29 (8.00–13.00)	10.80 ± 1.69 (8.00–14.00)	0.984
Disease duration (years) (minimum-maximum)	9.67 ± 6.02 (2.00–21.00)	N/A	N/A
LEDD (mg/day) (minimum-maximum)	802.54 ± 469.57 (0.00–1730.38)	N/A	N/A
Hoehn and Yahr stage^*^	3 (2.0–3.0)	0 (0.0–0.0)	**< 0.001**
CGI-S (minimum-maximum)	3.90 ± 0.70 (3.00–5.00)	1.93 ± 0.98 (1.00–4.00)	**< 0.001**
NMSQ	11.22 ± 5.65	2.67 ± 2.47	**< 0.001**
SCOPA-AUT Item 5^a^	0.90 ± 1.02	0.03 ± 0.18	**< 0.001**
SCOPA-AUT Item 6^b^	1.20 ± 1.06	0.23 ± 0.43	**< 0.001**
MoCA	27.00 ± 2.30	28.47 ± 1.53	**< 0.05**
BDI	9.55 ± 9.47	1.70 ± 2.61	**< 0.001**
PDQ-8	8.80 ± 5.19	N/A	N/A
EQ-5D-5L Index Value	0.76 ± 0.19	0.96 ± 0.06	**< 0.001**
pGDQ (minimum-maximum)	18.05 ±12.40 (0.00–40.00)	6.10 ± 3.11 (2.00–12.00)	**< 0.001**
Non-alcoholic drinks (ml/d) (minimum-maximum)	1411.90 ± 602.48 (500.00–2500.00)	1848.33 ± 666.63 (500.00–3750.00)	**< 0.05**
Caffeinated drinks (ml/d) (minimum-maximum)	485.71 ± 222.57 (0.00–800.00)	478.33 ± 307.29 (0.00–1500.00)	**< 0.05**
Alcoholic drinks (ml/d) (minimum-maximum)	100.68 ± 191.28 (0.00–750.00)	173.71 ± 331.85 (0.00–1500.00)	**< 0.05**

Data are presented as mean ± SD or ^*^median (25^*th*^-75^*th*^ percentiles). Differences between groups were tested using the Mann–Whitney U-test or Pearson chi-square test were appropriate. PD, Parkinson's disease; LEDD, Levodopa Equivalent Daily Dose; CGI-S, Clinical Global Impression–Severity scale; NMSQ, Non-Motor Symptoms Questionnaire; SCOPA-AUT, Scales for Outcomes in PD–Autonomic; MoCA, Montreal Cognitive Assessment; BDI, Beck Depression Inventory; PDQ-8, Parkinson's Disease Questionnaire-8; pGDQ, preliminary Gut Dysmotility Questionnaire. ^a^Item 5, “In the past month, have you had problems with constipation?” (0 = Never; 1 = Sometimes; 2 = Regularly; 3 = Often); ^b^Item 6, “In the past month, did you have to strain hard to pass stools?” (0 = Never; 1 = Sometimes; 2 = Regularly; 3 = Often). The *p*-values are bold if they are significant (< 0.05).

The neurologists (63.6% female patients) had a mean (±SD) age of 37.2 ± 11.4 (ranging from 27.5 to 66.6) years and a mean duration of experience in neurology of 8.6 ± 10.0 (ranging from 0.8 to 35.0) years with 45.5% acting as a resident physician and 54.5% as a consultant or in a higher position. In the total group, the years of experience, particularly in PD, were 5.6 ± 9.8 (ranging from: 0.0 to 30.0).

The included PwPD and HC were age- and sex-matched, and cognitive assessments were within normal ranges so that the results of self-completed questionnaires and scales were considered to be reliable (Table 1). Regarding data quality, no relevant data from any of the study participants were missing.

Patients with Parkinson's disease showed a significantly higher impairment in comparison to HC in all PD-specific questionnaires and scales evaluating motor and non-motor symptoms as well as in the clinical global impression of health state. Furthermore, PwPD presented with a significantly worse HRQoL in contrast to HC (Table 1).

**Figure 2:**
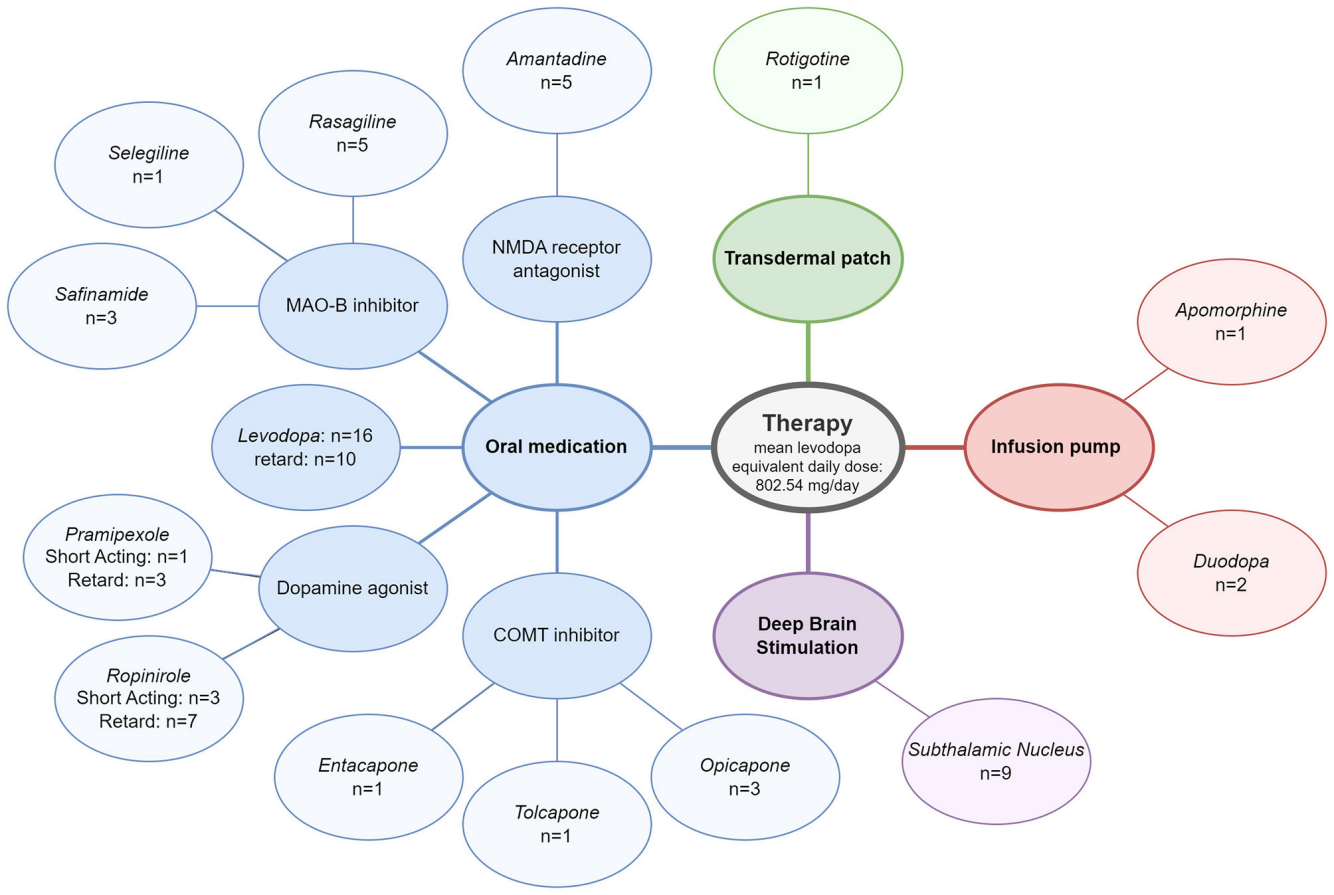
Therapy of patients with Parkinson's disease included in phase 2a.

Significant differences in the confounders and co-morbidities recorded were found between PwPD and HC, with PwPD presenting more often with depression (*p* < 0.01), dysphagia (*p* < 0.05), and surgery on the gastrointestinal tract (*p* < 0.01), especially the small/large intestine (*p* < 0.05). There were also significant differences in the use of antidepressants (*p* < 0.01), antipsychotics (*p* < 0.05), painkillers (*p* < 0.01), laxatives (*p* < 0.001), and ulcer therapy (*p* < 0.05), which were taken more frequently by PwPD. In addition, PwPD exercised less (*p* < 0.05) but got physiotherapy more often (*p* < 0.001) compared to HC. All PwPD received PD-specific therapy, of which 76.2% of patients received combination therapy of at least two drugs. Approximately, 28.6% of PwPD had an advanced therapy with deep brain stimulation and at least one oral medication, and 14.3% of patients used a pump therapy and at least one oral medication. An overview of all PD therapies in the PwPD group is provided in Figure 2.

Patients with Parkinson's disease showed a significantly higher total score in the pGDQ compared to HC. Furthermore, in five out of eight domains of the pGDQ, PwPD scored significantly higher than HC (Table 2). This is in correspondence with the results of validated measures of constipation in PD such as NMSQuest question 5 (percentage “yes-answer” in PwPD 57.1% vs. in HC 0%, *p* < 0.001, MW U-test) and SCOPA-AUT question 5 (percentage with constipation in PwPD 50% vs. in HC 3.3%, *p* < 0.01, chi-square test). The pGDQ total score, PD duration (*r*_*s*_ = 0.29, *p* > 0.05), and LEDD (*r*_*s*_ = 0.32, *p* > 0.05) showed a weak positive correlation.

**Table 2:** Total and domain scores and completion time of the preliminary GDQ of patients with Parkinson's disease and healthy controls of the phase 2a study.

**pGDQ**	**PD patients (*n =* 21)**	**Healthy controls (*n =* 30)**	***P*-value**
Total score	18.05 ± 12.40 (0.00 to 40.00)	6.10 ± 3.11 (2.00 to 12.00)	**< 0.001**
Domain-frequency	1.43 ± 1.40 (0.00 to 4.00)	0.00 ± 0.00 (0.00 to 0.00)	**< 0.001**
Domain-duration	2.14 ± 1.46 (0.00 to 5.00)	0.27 ± 0.45 (0.00 to 1.00)	**< 0.001**
Domain-severity	4.14 ± 2.67 (0.00 to 11.00)	1.27 ± 1.05 (0.00 to 4.00)	**< 0.001**
Domain-consistency	4.24 ± 2.90 (0.00 to 9.00)	3.10 ± 1.45 (0.00 to 5.00)	0.096
Domain-assistance	1.24 ± 1.51 (0.00 to 5.00)	0.10 ± 0.55 (0.00 to 3.00)	**< 0.001**
Domain-pain	3.48 ± 3.39 (0.00 to 10.00)	1.57 ± 1.48 (0.00 to 6.00)	0.056
Domain-HRQoL	1.67 ± 1.59 (0.00 to 5.00)	0.23 ± 0.57 (0.00 to 2.00)	**< 0.001**
Domain-development	−0.35 ± 1.06 (-4.00 to 1.00)	−1.30 ± 2.11 (-5.00 to 0.00)	0.505
Completion time (min)	8.45 ± 5.28 (3.00 to 15.00)	5.90 ± 2.24 (2.00 to 5.00)	**< 0.05**

Data are presented as mean ± SD (minimum–maximum). PD, Parkinson's Disease; pGDQ, preliminary Gut Dysmotility Questionnaire; HRQoL, Health-Related Quality of Life. The *p*-values are bold if they are significant (< 0.05).

##### 3.2.1.2. Acceptability

The GDQ total score showed a minor floor effect with 4.8% of PwPD having the lowest total score, but no ceiling effect. The pGDQ domains showed a moderate floor effect, ranging from 4.8% of PwPD reaching the lowest score in the domain severity up to 52.4% in the domain assistance and from 3.3% of HC reaching the lowest score in the domain development up to 100% in the domain frequency. None of the pGDQ domains showed a ceiling effect. Apart from the assistance domain (5.48) in the HC, moderate skewness was found for all domains and the total score in both groups.

##### 3.2.1.3. Psychometric properties

Internal consistency was high for all items of the questionnaire (Cronbach's alpha value of 0.92), and for the domain pain (α = 0.92), it was good for the domain frequency (α = 0.75) and adequate for all other domains (α = 0.46–0.68) in PwPD.

The intercorrelation and construct validity of the pGDQ are summarized in Table 3. In PwPD, all pGDQ domains, except development, showed a high-level positive correlation with the total score (*r*_*s*_ = 0.67–0.91; *p* < 0.001). The pGDQ domains showed a moderate- to high-level positive correlation with each other (*r*_*s*_ = 0.44–0.91, *p* < 0.05) apart from a weak positive correlation between the domain pain and frequency (*r*_*s*_ = 0.30, *p* > 0.05) and any correlation of the domain development. The total score of the pGDQ correlated positively on a high level with the NMSQ total score and Item five as well as Item seven of the NMSQ, which are specific to assess constipation. It is noteworthy that the pGDQ total score also correlated on a high level with the NMSQ Items 12 and 13, which relate to memory and mood. The SCOPA-AUT Item five, Item six, and the total score as well as the MDS-UPDRS Item 1.11 and the PDQ-8 total score correlated positively on a high level with the pGDQ total score (Table 3). The total score of the pGDQ also correlated positively on a moderate level with the Hoehn and Yahr stage, with the BDI, and on a weak level with the CGI-S (Table 3). The PDQ-8 total score correlated positively on a high level with the pGDQ QoL domain.

**Table 3:** Intercorrelation and construct validity of pGDQ domains in patients with Parkinson's disease.

	**pGDQ-total score**	**pGDQ-frequency**	**pGDQ-duration**	**pGDQ-severity**	**pGDQ-consistency**	**pGDQ-assistance**	**pGDQ-pain**	**pGDQ-HRQoL**	**pGDQ-development**
pGDQ-frequency	0.79^***^								
pGDQ-duration	0.81^***^	0.70^***^							
pGDQ-severity	0.88^***^	0.72^***^	0.66^**^						
pGDQ-consistency	0.84^***^	0.55^**^	0.57^**^	0.61^**^					
pGDQ-assistance	0.79^***^	0.64^**^	0.82^***^	0.66^**^	0.53^**^				
pGDQ-pain	0.67^***^	0.30	0.50^*^	0.58^**^	0.68^***^	0.44^*^			
pGDQ-HRQoL	0.91^***^	0.63^**^	0.79^***^	0.81^***^	0.66^**^	0.84^***^	0.64^**^		
pGDQ-development	−0.05	−0.04	−0.24	−0.09	−0.08	−0.06	0.04	0.07	
Hoehn and Yahr stage	0.56^**^	0.60^**^	0.61^**^	0.44^*^	0.48^*^	0.46^*^	0.22	0.41^*^	0.32
CGI-S	0.37^*^	0.67^***^	0.58^**^	0.31	0.20	0.34	0.18	0.33	−0.02
NMSQ-item 5^a^	0.80^***^	0.84^***^	0.77^***^	0.66^**^	0.61^**^	0.76^***^	0.32	0.74^***^	0.07
NMSQ-item 7^b^	0.76^***^	0.77^***^	0.72^***^	0.71^***^	0.58^**^	0.65^**^	0.54^*^	0.66^**^	−0.14
NMSQ-item 12^c^	0.70^**^	0.47^*^	0.71^**^	0.71^**^	0.48^*^	0.53^*^	0.47^*^	0.70^**^	−0.15
NMSQ-item 13^d^	0.71^***^	0.75^***^	0.60^**^	0.58^**^	0.66^**^	0.45^*^	0.65^**^	0.52^*^	−0.19
NMSQ-total score	0.85^***^	0.64^**^	0.81^***^	0.84^***^	0.71^***^	0.64^**^	0.62^**^	0.74^***^	−0.15
SCOPA-AUT-item 5^e^	0.62^**^	0.71^***^	0.73^***^	0.45^*^	0.48^*^	0.65^**^	0.26	0.56^**^	0.03
SCOPA-AUT-item 6^f^	0.77^***^	0.76^***^	0.79^***^	0.66^**^	0.55^**^	0.70^***^	0.46^*^	0.72^***^	0.05
SCOPA-AUT-total score	0.75^***^	0.80^***^	0.78^***^	0.64^**^	0.63^**^	0.63^**^	0.43	0.65^**^	−0.03
MDS-UPDRS -item 1.11^g^	0.72^***^	0.49^*^	0.71^***^	0.58^**^	0.58^**^	0.75^***^	0.61^**^	0.69^**^	−0.16
PDQ-8-total score	0.83^***^	0.77^***^	0.77^***^	0.77^***^	0.64^**^	0.65^**^	0.59^**^	0.80^***^	−0.01
BDI-total score	0.57^**^	0.60^**^	0.62^**^	0.54^**^	0.50^*^	0.33	0.36	0.45^*^	−0.07

Spearman's rank correlation coefficients. ^*^P < 0.05; ^**^P < 0.01; ^***^P < 0.001. BDI, Beck Depression Inventory; CGI-S, Clinical Global Impression–Severity scale; MDS-UPDRS, Movement Disorder Society-Unified Parkinson's Disease Rating Scale, NMSQ, Non-Motor Symptoms Questionnaire; PDQ-Q, Parkinson's Disease Questionnaire; pGDQ, preliminary Gut Dysmotility Questionnaire; HRQoL, Health-Related Quality of Life; SCOPA-AUT, Scales for Outcomes in PD–Autonomic. ^a^Item 5, “Have you experienced any of the following in the last month? Constipation (< 3 bowel movements a week) or having to strain to pass a stool (feces)” (0 = No; 1 = Yes); ^b^Item 7, “Have you experienced any of the following in the last month? Feeling that your bowel emptying is incomplete after having been to the toilet” (0 = No; 1 = Yes); ^c^Item 12, “Have you experienced any of the following in the last month? Problems remembering things that have happened recently or forgetting to do things” (0 = No; 1 = Yes); ^d^Item 13, “Have you experienced any of the following in the last month? Loss of interest in what is happening around you or doing things” (0 = No; 1 = Yes); ^*e*^Item 5, “In the past month, have you had problems with constipation?” (0 = Never; 1 = Sometimes; 2 = Regularly; 3 = Often); ^f^Item 6, “In the past month, did you have to strain hard to pass stools?” (0 = Never; 1 = Sometimes; 2 = Regularly; 3 = Often); ^g^Item 1.11, “Over the past week have you had constipation troubles that cause you difficulty moving your bowels?” (0 = Normal; 1 = Slight; 2 = Mild; 3 = Moderate; 4 = Severe).

In HC, the total score of the pGDQ correlated positively with the NMSQ on a weak level (*r*_*s*_ = 0.33, *p* < 0.05). In addition, the QoL domain of the pGDQ correlated negatively on a weak level with the EQ-5D-5L score (*r*_*s*_ = −0.43, *p* < 0.01) (Table 3).

##### 3.2.1.4. Evaluation of the pGDQ using the interview protocol and the evaluation questionnaire with corresponding adaptation

In total, 355 problems were identified in the interviews with PwPD and HC which were performed directly after the self-completion of the pGDQ. These problems were categorized into 24 CCS codes, which were assigned to the corresponding questions of the pGDQ (Table 4). In particular, question 16 with overall 27% entries, question 12 with 17.5%, question 2 with 7.3%, and question 8.1 with 6.5% entries were found to stand out. The highest-rated issues were the type of answer possibilities with “unclear respondent instruction” and “missing response categories” for questions 12 (stool consistency) and 16 (development of constipation during the past 3 months). In both questions, the answer options were differently designed compared to the four-item response scale of most other questions, which was well received. Therefore, in question 12, the type of answer option was changed from a table to individual questions with the four-item response scale. Moreover, the visual analog scale of question 16, which was just a line without any numeric values was adapted comprising boxes ranging from “constipation gets worse” (-5 points) to “no change in constipation” (0 points) to “constipation gets better” (5 points), and one further box has an alternative answer option of “no constipation.” Hereby, also the scoring of the answer was improved as it had been prone to errors in the evaluation by neurologists with a relevant number of total scores being incorrectly calculated. In addition, question 16 was excluded to be counted toward the total score of the GDQ based on results of the intercorrelation and convergent validity but was retained in the questionnaire as it was found to be valuable by neurologists. Another often observed issue was “complex/awkward syntax” for questions 2 (duration of constipation in years) and 6 (incomplete evacuation). Therefore, the wording of question 2 was simplified. Question 6 was removed from the questionnaire due to the results of the evaluation questionnaire, which showed no meaningful difference between questions 5 and 6. Question 5 remained as it was better received and evaluated. “Complex/awkwardly detailed response definition” was a common issue for many questions. Questions 3 (straining during defecation), 4 (constriction in the anus during defecation), 5 (incomplete evacuation), 6 (incomplete evacuation), 8 (painful abdomen), 9 (rectal pain), 10 (laxative usage), 11 (manual aid for defecation), 12 (stool consistency), and 13 (fecal incontinence) had frequencies as response options with additional text in brackets to specify the terms, which was often found to be confusing or too detailed. In addition, PwPD and HC did not find the answer options to be exhaustive as rated in “missing response categories.” There was a lack of options, e.g., in frequency-related response options, such as “rarely” between the provided choices “never” and “sometimes.” Subsequently, all frequency response options were replaced with the four-item response scale “never,” “rarely/sometimes,” “often,” and “mostly/always.” The response options of question 15 (quality of life) were found to be too complex and were simplified. Questions 8.2 and 9.2 used severity response options with definitions in brackets, which were found to be too detailed and confusing. The answer options were simplified to “not applicable,” “mild,” “moderate,” and “severe.” Another point of criticism was the conditional omission of questions. This applied to the four questions with subcategories of frequency and severity: question 8 about painful pull in the stomach or unpleasant bloating, 9 about rectal pain, 10 about the use of laxatives, and 13 about stool incontinence. If the frequency “never” was chosen, the question about the severity should be skipped. This was confusing as well as it was not followed by some participants and therefore caused an incorrect scoring of the pGDQ. As a consequence, the subcategories of questions 8 and 9 were changed to two different questions, one asking for frequency and one for severity. Question 10 was reduced to one question, not asking about the efficacy of the use of laxatives anymore. Question 13 on fecal incontinence was removed from the questionnaire due to an additional low inter-item correlation in its domain, and it reduced the internal consistency of the questionnaire measurably.

**Table 4:** Problem labels for the classification coding scheme codes of each question of the pGDQ compiled by the interview protocols of patients with Parkinson's disease and healthy controls for the phase 2a study.

	**Question number of the preliminary Gut Dysmotility Questionnaire [frequency (** ***N*** **), occurrence per question in %]**																																								**Total (frequency, overall in %)**	
	**1**		**2**		**3**		**4**		**5**		**6**		**7**		**8.1**		**8.2**		**9.1**		**9.2**		**10.1**		**10.2**		**11**		**12**		**13.1**		**13.2**		**14**		**15**		**16**			
	***N***	***%***	***N***	***%***	***N***	***%***	***N***	***%***	***N***	***%***	***N***	***%***	***N***	***%***	***N***	***%***	***N***	***%***	***N***	***%***	***N***	***%***	***N***	***%***	***N***	***%***	***N***	***%***	***N***	***%***	***N***	***%***	***N***	***%***	***N***	***%***	***N***	***%***	***N***	***%***	***N***	***%***
Complex estimation, difficult mental calculation required	0	0.0%	0	0.0%	0	0.0%	0	0.0%	0	0.0%	0	0.0%	0	0.0%	0	0.0%	0	0.0%	0	0.0%	0	0.0%	0	0.0%	0	0.0%	0	0.0%	2	3.2%	0	0.0%	0	0.0%	0	0.0%	0	0.0%	1	1.0%	3	0.8%
Complex topic	0	0.0%	0	0.0%	0	0.0%	0	0.0%	0	0.0%	0	0.0%	0	0.0%	0	0.0%	0	0.0%	0	0.0%	0	0.0%	0	0.0%	0	0.0%	0	0.0%	1	1.6%	0	0.0%	0	0.0%	0	0.0%	0	0.0%	0	0.0%	1	0.3%
Complex/awkward syntax	0	0.0%	12	46.2%	0	0.0%	0	0.0%	0	0.0%	7	43.8%	0	0.0%	0	0.0%	0	0.0%	0	0.0%	0	0.0%	0	0.0%	0	0.0%	0	0.0%	0	0.0%	0	0.0%	0	0.0%	0	0.0%	3	23.1%	0	0.0%	22	6.2%
Complex/awkwardly detailed response definition	1	12.5%	0	0.0%	9	50.0%	6	33.3%	5	35.7%	2	12.5%	0	0.0%	5	21.7%	1	25.0%	6	50.0%	0	0.0%	2	33.3%	1	12.5%	3	50.0%	6	9.7%	2	40.0%	0	0.0%	0	0.0%	1	7.7%	2	2.1%	52	14.6%
Erroneous assumption	0	0.0%	0	0.0%	0	0.0%	0	0.0%	0	0.0%	0	0.0%	1	33.3%	0	0.0%	0	0.0%	0	0.0%	0	0.0%	0	0.0%	0	0.0%	0	0.0%	1	1.6%	0	0.0%	0	0.0%	0	0.0%	0	0.0%	2	2.1%	4	1.1%
High detail required or information unavailable	0	0.0%	0	0.0%	0	0.0%	0	0.0%	0	0.0%	0	0.0%	1	33.3%	0	0.0%	0	0.0%	0	0.0%	0	0.0%	0	0.0%	0	0.0%	0	0.0%	5	8.1%	0	0.0%	0	0.0%	0	0.0%	0	0.0%	0	0.0%	6	1.7%
Layout or formatting	0	0.0%	0	0.0%	0	0.0%	0	0.0%	0	0.0%	0	0.0%	0	0.0%	0	0.0%	0	0.0%	0	0.0%	0	0.0%	0	0.0%	0	0.0%	0	0.0%	7	11.3%	0	0.0%	0	0.0%	0	0.0%	0	0.0%	6	6.3%	13	3.7%
Long recall or reference period	3	37.5%	5	19.2%	0	0.0%	0	0.0%	1	7.1%	2	12.5%	0	0.0%	0	0.0%	0	0.0%	0	0.0%	0	0.0%	0	0.0%	0	0.0%	0	0.0%	1	1.6%	0	0.0%	0	0.0%	0	0.0%	0	0.0%	0	0.0%	12	3.4%
Missing response categories	0	0.0%	1	3.8%	5	27.8%	2	11.1%	2	14.3%	0	0.0%	0	0.0%	4	17.4%	0	0.0%	1	8.3%	0	0.0%	0	0.0%	0	0.0%	0	0.0%	1	1.6%	0	0.0%	0	0.0%	1	33.3%	0	0.0%	30	31.3%	47	13.2%
Non-verbal reaction (re-reading, skeptical or thoughtful)	2	25.0%	1	3.8%	0	0.0%	0	0.0%	2	14.3%	1	6.3%	0	0.0%	1	4.3%	0	0.0%	0	0.0%	0	0.0%	0	0.0%	1	12.5%	0	0.0%	4	6.5%	0	0.0%	0	0.0%	0	0.0%	2	15.4%	9	9.4%	23	6.5%
Other answer type preferred	1	12.5%	0	0.0%	0	0.0%	0	0.0%	0	0.0%	0	0.0%	0	0.0%	0	0.0%	0	0.0%	0	0.0%	0	0.0%	0	0.0%	0	0.0%	0	0.0%	5	8.1%	0	0.0%	0	0.0%	0	0.0%	1	7.7%	2	2.1%	9	2.5%
Overlapping categories	0	0.0%	0	0.0%	0	0.0%	0	0.0%	0	0.0%	0	0.0%	0	0.0%	0	0.0%	0	0.0%	0	0.0%	0	0.0%	0	0.0%	0	0.0%	0	0.0%	1	1.6%	0	0.0%	0	0.0%	1	33.3%	0	0.0%	0	0.0%	2	0.6%
Potentially sensitive or desirability bias	0	0.0%	0	0.0%	1	5.6%	0	0.0%	0	0.0%	0	0.0%	0	0.0%	8	34.8%	0	0.0%	0	0.0%	0	0.0%	0	0.0%	0	0.0%	1	16.7%	1	1.6%	0	0.0%	0	0.0%	0	0.0%	0	0.0%	0	0.0%	11	3.1%
Question not applicable to some respondents	0	0.0%	0	0.0%	0	0.0%	0	0.0%	0	0.0%	0	0.0%	0	0.0%	0	0.0%	0	0.0%	0	0.0%	0	0.0%	0	0.0%	0	0.0%	0	0.0%	0	0.0%	0	0.0%	0	0.0%	0	0.0%	0	0.0%	32	33.3%	32	9.0%
Question order	0	0.0%	0	0.0%	0	0.0%	0	0.0%	0	0.0%	0	0.0%	0	0.0%	0	0.0%	0	0.0%	0	0.0%	0	0.0%	0	0.0%	0	0.0%	0	0.0%	0	0.0%	0	0.0%	0	0.0%	0	0.0%	0	0.0%	2	2.1%	2	0.6%
Question too long	0	0.0%	0	0.0%	0	0.0%	0	0.0%	0	0.0%	0	0.0%	0	0.0%	0	0.0%	0	0.0%	0	0.0%	0	0.0%	0	0.0%	0	0.0%	0	0.0%	0	0.0%	0	0.0%	0	0.0%	0	0.0%	4	30.8%	0	0.0%	4	1.1%
Several questions	0	0.0%	0	0.0%	0	0.0%	0	0.0%	0	0.0%	0	0.0%	0	0.0%	0	0.0%	0	0.0%	0	0.0%	0	0.0%	0	0.0%	0	0.0%	0	0.0%	1	1.6%	0	0.0%	0	0.0%	0	0.0%	0	0.0%	0	0.0%	1	0.3%
Topic carried over from earlier question	0	0.0%	0	0.0%	0	0.0%	1	5.6%	0	0.0%	0	0.0%	0	0.0%	0	0.0%	0	0.0%	0	0.0%	0	0.0%	0	0.0%	0	0.0%	0	0.0%	0	0.0%	0	0.0%	0	0.0%	0	0.0%	2	15.4%	0	0.0%	3	0.8%
Uncertain or failure to skip	0	0.0%	0	0.0%	0	0.0%	0	0.0%	0	0.0%	0	0.0%	0	0.0%	0	0.0%	0	0.0%	0	0.0%	5	83.3%	0	0.0%	6	75.0%	0	0.0%	0	0.0%	0	0.0%	2	25.0%	0	0.0%	0	0.0%	0	0.0%	13	3.7%
Unclear respondent instruction	0	0.0%	0	0.0%	0	0.0%	0	0.0%	0	0.0%	0	0.0%	0	0.0%	0	0.0%	0	0.0%	0	0.0%	0	0.0%	0	0.0%	0	0.0%	0	0.0%	25	40.3%	0	0.0%	0	0.0%	0	0.0%	0	0.0%	7	7.3%	32	9.0%
Undefined term	0	0.0%	2	7.7%	0	0.0%	0	0.0%	0	0.0%	0	0.0%	1	33.3%	0	0.0%	1	25.0%	0	0.0%	1	16.7%	0	0.0%	0	0.0%	1	16.7%	0	0.0%	1	20.0%	1	12.5%	0	0.0%	0	0.0%	0	0.0%	8	2.3%
Undefined/vague term	1	12.5%	1	3.8%	0	0.0%	8	44.4%	3	21.4%	0	0.0%	0	0.0%	3	13.0%	1	25.0%	5	41.7%	0	0.0%	4	66.7%	0	0.0%	1	16.7%	0	0.0%	1	20.0%	0	0.0%	0	0.0%	0	0.0%	0	0.0%	28	7.9%
Vague term	0	0.0%	1	3.8%	1	5.6%	1	5.6%	0	0.0%	1	6.3%	0	0.0%	0	0.0%	0	0.0%	0	0.0%	0	0.0%	0	0.0%	0	0.0%	0	0.0%	0	0.0%	0	0.0%	5	62.5%	0	0.0%	0	0.0%	0	0.0%	9	2.5%
Vague/unclear question	0	0.0%	3	11.5%	2	11.1%	0	0.0%	1	7.1%	3	18.8%	0	0.0%	2	8.7%	1	25.0%	0	0.0%	0	0.0%	0	0.0%	0	0.0%	0	0.0%	1	1.6%	1	20.0%	0	0.0%	1	33.3%	0	0.0%	3	3.1%	18	5.1%
Total (frequency, occurrence overall in %)	8	2.3%	26	7.3%	18	5.1%	18	5.1%	14	3.9%	16	4.5%	3	0.8%	23	6.5%	4	1.1%	12	3.4%	6	1.7%	6	1.7%	8	2.3%	6	1.7%	62	17.5%	5	1.4%	8	2.3%	3	0.8%	13	3.7%	96	27.0%	355	100.0%

The results of the evaluation questionnaires of PwPD, HC, and neurologists are presented in Table 5. Most study participants of the three groups found the pGDQ to be relevant and helpful to assess current gastrointestinal health state, comprehensive, simple, and clear to understand; to be having suitable, clear, and appropriate answers; and to be having a sensible order of the questions. About half of the study participants of each group found the pGDQ to be difficult to answer. This was in line with the results of the interview protocol as described above. Disagreement was found in the question if the pGDQ is too long with 54.5% of the neurologists evaluating the pGDQ as too long in contrast to PwPD (23.8 %) and HC (3.3 %) who are the once who completed the pGDQ. Due to the removal of questions and streamlining of the pGDQ by simplification as described above, we addressed this issue. Interestingly, 27.3% of the neurologists found the pGDQ strange or embarrassing whereas none of the HC and only 14.3 % of the PwPD declared this.

**Table 5:** Results of the evaluation questionnaire for neurologists and patients with Parkinson's disease of the phase 2a and 2b study.

**Questions of the evaluation questionnaire**	**Answer options**	**Neurologists**				**PD patients**			
		**Phase 2a**		**Phase 2b**		**Phase 2a**		**Phase 2b**	
		**(*****n*** = **11)**		**(*****n*** = **5)**		**(*****n*** = **21)**		**(*****n*** = **10)**	
		***N***	***%***	***N***	***%***	***N***	***%***	***N***	***%***
Do you consider the questionnaire relevant?	Yes	11	100.0	5	100.0	19	90.5	10	100.0
	No	0	0.0	0	0.0	2	9.5	0	0.0
Does the questionnaire help you to assess the current health status related to gastrointestinal symptoms of your PD patients?	Yes	11	100.0	5	100.0	16	76.2	9	90.0
	No	0	0.0	0	0.0	5	23.8	1	10.0
Do you find the questionnaire sufficiently comprehensive?	Yes	11	100.0	5	100.0	17	81.0	9	90.0
	No	0	0.0	0	0.0	4	19.0	0	0.0
Do you think the questionnaire is too long?	Yes	6	54.5	1	20.0	5	23.8	0	0.0
	No	5	45.5	4	80.0	16	76.2	10	100.0
Do you find the questions simple and clear to understand?	Yes	8	72.7	5	100.0	13	61.9	9	90.0
	No	3	27.3	0	0.0	8	38.1	1	10.0
Do you find questions strange / embarrassing?	Yes	3	27.3	N/A		3	14.3	0	0.0
	No	8	72.7	N/A		18	85.7	10	100.0
Do you find certain questions difficult to answer?	Yes	5	45.5	N/A		11	52.4	1	10.0
	No	6	54.5	N/A		10	47.6	9	90.0
Do you find the answer options suitable, clear and appropriate?	Yes	9	81.8	5	100.0	15	71.4	9	90.0
	No	2	18.2	0	0.0	6	28.6	0	0.0
Do you find the order of the questions sensible?	Yes	10	90.9	5	100.0	21	100	9	90.0
	No	1	9.1	0	0.0	0	0.0	0	0.0
Do you have any comments or general suggestions for improving the questionnaire?	Yes	6	54.5	N/A		5	23.8	3	30.0
	No	5	45.5	N/A		16	76.2	7	70.0
Do you find the instructions for conducting and evaluating the questionnaire suitable?	Yes	N/A		5	100.0	N/A		N/A	
	No	N/A		0		N/A		N/A	
Does the questionnaire help you in screening healthy controls for gastrointestinal symptoms?	Yes	10	90.9	5	100.0	N/A		N/A	
	No	1	9.1	0	0.0	N/A		N/A	
Do you find the evaluation of the questionnaire suitable?	Yes	6	54.5	5	100.0	N/A		N/A	
	No	5	45.5	0	0.0	N/A		N/A	
Do you find the assignment of the individual questions to the 8 different domains correct and sensible?	Yes	8	72.7	5	100.0	N/A		N/A	
	No	3	27.3	0	0.0	N/A		N/A	

PD, Parkinson's disease.

The evaluation questionnaires of the neurologists revealed that the scoring of the pGDQ was too complex, mainly due to the different types as well as the changing value of the response options (from low to high and high to low scores). As the response options were homogenized based on the feedback by the PwPD and HC in the interviews as shown earlier, the scoring got simplified. In addition, all response options were scored from left to right with increasing scores.

Based on these results of the phase 2a study, the preliminary GDQ was adapted to the pfGDQ, which was tested in a phase 2b study. The pfGDQ consisted of only 18 instead of 24 questions and did not contain any sub-questions. The questions were still assigned to the same eight domains as in the pGDQ (Table 2). All answers were provided on a four-item response scale, which was equalized wherever possible. Only the answer option of the domain development remained as a visual analog scale in an adapted version as described above.

#### 3.2.2. Phase 2b study: Cognitive pretests of the prefinal GDQ

In phase 2b, the adapted pGDQ, titled pfGDQ, was cognitively pretested in a smaller sample size to evaluate the changes and to create the final GDQ. A total of 10 PwPD, 10 HC, and five neurologists were investigated.

Demographic, motor, and non-motor characteristics of PwPD and HC are summarized in Table 6.

**Table 6:** Demographic, motor, and non-motor characteristics and prefinal GDQ score characteristics of patients with Parkinson's disease and healthy controls of the phase 2b study.

	**PD patients (*n =* 10)**	**Healthy controls (*n =* 10)**	***P* value**
Age (years) (minimum-maximum)	67.80 ± 9.51 (47.00 to 82.00)	64.41 ± 14.30 (32.00 to 80.00)	0.631
Sex (m/f)	4/6	5/5	0.653
Disease duration (years) (minimum-maximum)	9.03 ± 5.80 (1.67 to 19.68)	N/A	N/A
Hoehn and Yahr stage^*^	2 (2.0 to 3.0)	0 (0.0 to 0.0)	**< 0.001**
MoCA (minimum-maximum)	27.17 ± 2.79 (22.00 to 30.00)	N/A	N/A
pfGDQ total score (minimum-maximum)	17.10 ± 9.92 (1.00 to 32.00)	6.40 ± 4.20 (1.00 to 13.00)	**< 0.05**
pfGDQ-frequency (minimum-maximum)	0.70 ± 0.48 (0.00 to 1.00)	0.00 ± 0.00 (0.00 to 0.00)	**< 0.01**
pfGDQ-duration (minimum-maximum)	1.40 ± 1.07 (0.00 to 3.00)	0.20 ± 0.42 (0.00 to 1.00)	**< 0.05**
pfGDQ-severity (minimum-maximum)	4.40 ± 2.84 (1.00 to 9.00)	1.50 ± 1.08 (0.00 to 3.00)	**< 0.05**
pfGDQ-consistency (minimum-maximum)	3.70 ± 2.36 (0.00 to 8.00)	1.80 ± 1.69 (0.00 to 4.00)	0.054
pfGDQ-assistance (minimum-maximum)	1.00 ± 1.15 (0.00 to 3.00)	0.00 ± 0.00 (0.00 to 0.00)	**< 0.01**
pfGDQ-pain (minimum-maximum)	4.10 ± 3.00 (0.00 to 8.00)	2.20 ± 1.69 (0.00 to 6.00)	0.135
pfGDQ-HRQoL (minimum-maximum)	1.80 ± 1.62 (0.00 to 4.00)	0.70 ± 1.06 (0.00 to 3.00)	0.278
pfGDQ-development (minimum-maximum)	−1.00 ± 3.16 (-5.00 to 5.00)	0.00 ± 0.00 (0.00 to 0.00)	**< 0.05**
pfGDQ-completion time (min)	7.40 ± 3.84	3.38 ± 0.98	**< 0.05**

Data are presented as mean ± SD or ^*^median (25^*th*^-75^*th*^ percentiles). Differences between groups were tested using the Mann–Whitney U-test or Pearson chi-square where appropriate. PD, Parkinson's disease; pfGDQ, prefinal Gut Dysmotility Questionnaire; MoCA, Montreal Cognitive Assessment; HRQoL, Quality of Life. The *p*-values are bold if they are significant (< 0.05).

The five neurologists (60% men), which also participated in phase 2a, were selected based on their answers of the evaluation questionnaire from phase 2a. Particular concern was given to those who were critical and who had negative comments. Their mean (±SD) age was 43.9 ± 14.7 (ranging from: 29.7 to 67.7) years, and their mean duration of general experience in neurology was 14.4 ± 5.6 (ranging from 3.0 to 35.0) years with 11.8 ± 5.5 years of experience particularly in PD.

Patients with Parkinson's disease and HC were matched for age and sex, and the cognitive scores were within normal ranges, so that the results of the self-completed questionnaires and scales were regarded as reliable (Table 6). Regarding data quality, one pfGDQ from a PwPD was incomplete and could not be used for full statistical analysis (missing 5%).

Patients with Parkinson's disease showed a significantly higher total score of the pfGDQ compared to HC. In addition, PwPD scored significantly higher in five out of the eight domains of the pfGDQ compared to HC (Table 6). The mean completion time of the pfGDQ was significantly longer for PwPD than for HC but shorter compared to the completion time of the pGDQ (in PwPD 1.05 and in HC 2.52 min less).

##### 3.2.2.1. Acceptability

The pfGDQ total score showed no floor and no ceiling effect. The pfGDQ domains showed a moderate floor effect, ranging from 10% of PwPD reaching the lowest score in the domain consistency up to 40% in the domain assistance and from 20% of HC reaching the lowest score in the domain severity, pain and development up to 100% in the domain frequency, assistance, and development. A low ceiling effect was detected with 10% of PwPD reaching the highest score in the domain severity and development. A moderate skewness was found for all domains and the total score in both groups.

##### 3.2.2.2. Psychometric properties

Internal consistency was high for all items of the pfGDQ (Cronbach's alpha value of 0.94). Further analyses were not performed as results of phase 2a were satisfying and the sample size of phase 2b was too small to result in any relevant new findings.

##### 3.2.2.3. Evaluation of the pfGDQ by the evaluation questionnaire with corresponding adaptation

The results of the evaluation questionnaires of the pfGDQ as assessed by PwPD, HC, and neurologists are summarized in Table 5. The majority of the three groups found the pfGDQ easy to understand, not too long, comprehensive, and relevant. There were no major points of criticism in the evaluation questionnaires of all three groups. The simplified scoring of the pfGDQ was an improvement as evaluated by the neurologists and reflected in zero errors in the calculation of the pfGDQ scores by the neurologists. Therefore, only minor adjustments to the pfGDQ were necessary. A grammatical error in the answer options of question 2 (duration) was criticized and corrected. Questions 14 and 15 (consistency) contained a description of consistency in parentheses, which was criticized as being too restrictive. To mitigate this, “for example” was added. Question 18 (development) also contained definition text in parentheses, which was removed.

**Figure 3:**
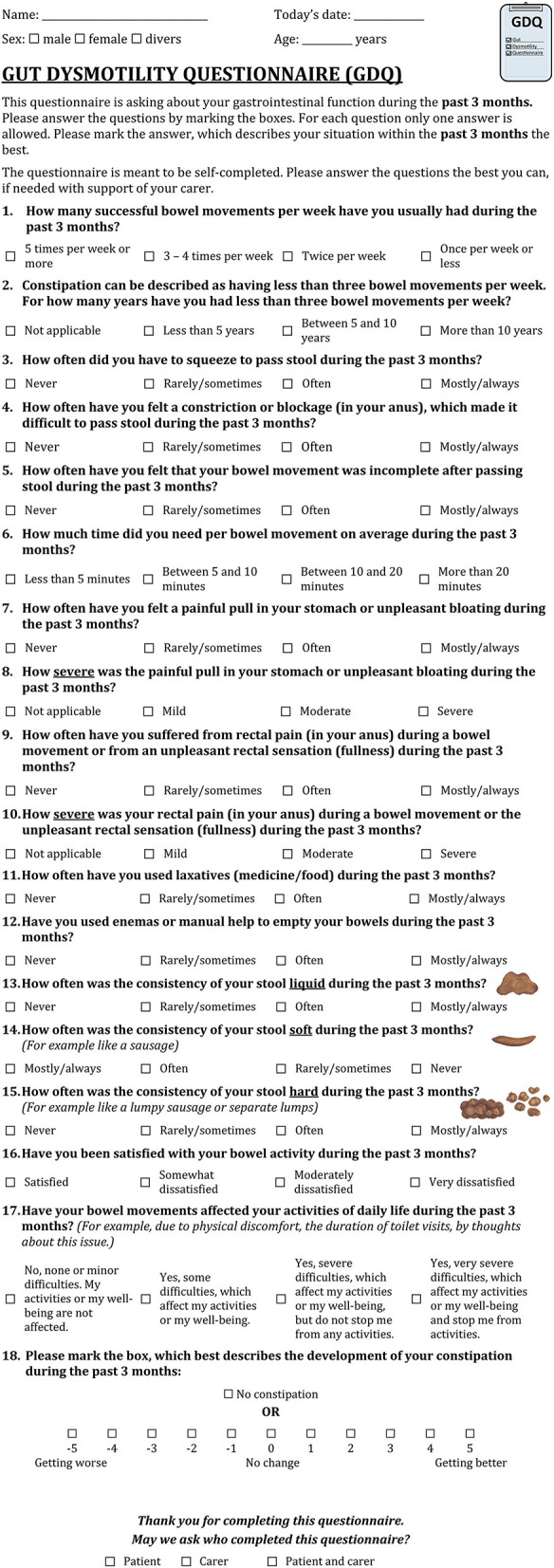
Gut Dysmotility Questionnaire (GDQ).

**Figure 4:**
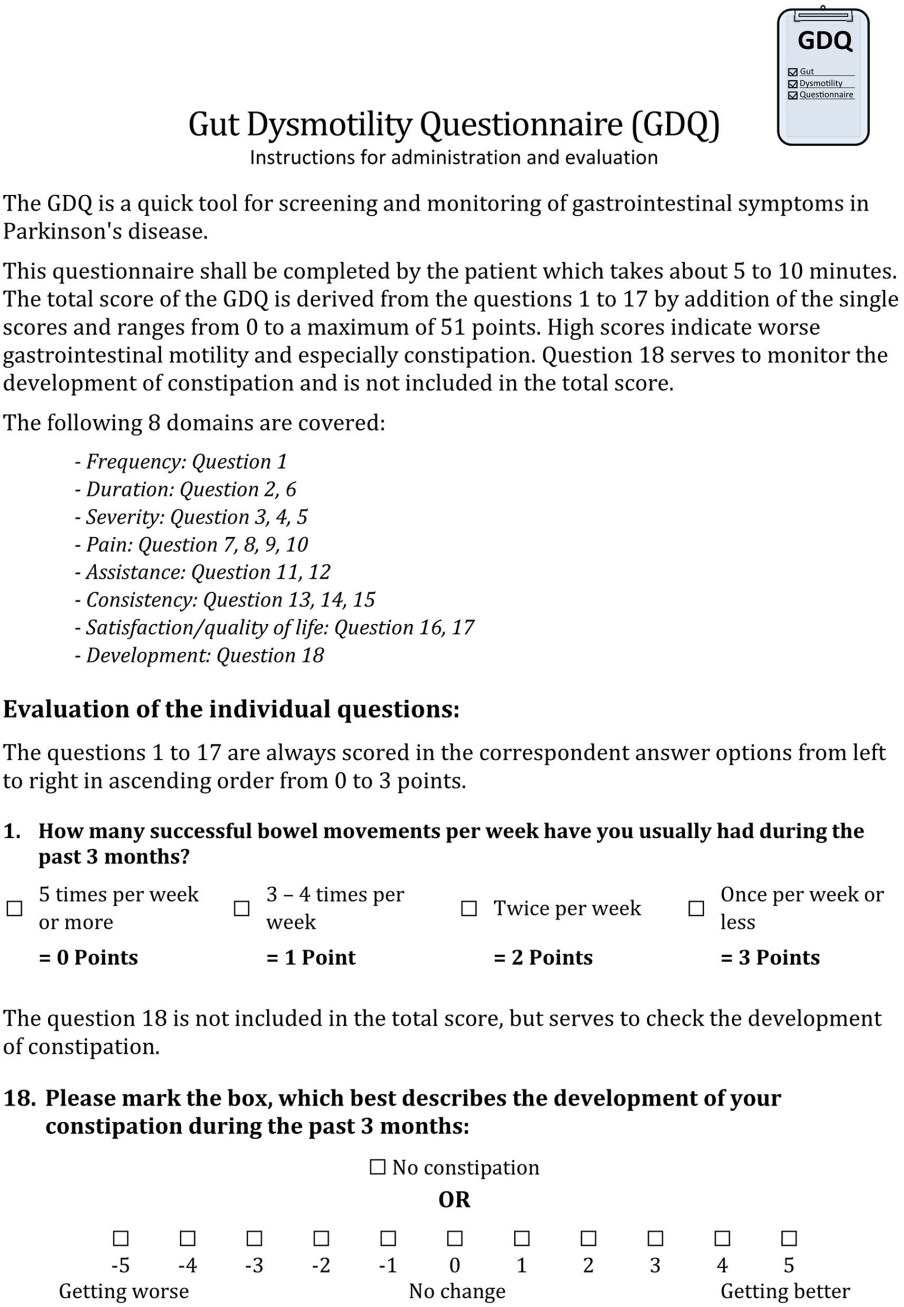
Instructions for administration and evaluation of the Gut Dysmotility Questionnaire (GDQ).

The phase 2b study resulted in the adaptation of the pfGDQ to the final GDQ. The final GDQ is a self-completed questionnaire consisting of 18 multiple-choice questions and takes approximately 4 min to complete (Figure 3, print version of the GDQ in Supplementary Figure 1). It covers eight domains (Table 7). The total score of the final GDQ results from the sum of the questions 1 to 17; each scored from 0 to 3 points from left to right in the respective answer options (Figure 4). The total score of the final GDQ accounts from 0 to a maximum of 51 points with higher scores indicating more disturbed gastrointestinal motility and, in particular, constipation. Question 18 is used to monitor the development of constipation and is not included in the total score. If there is a worsening of constipation, the score is increasingly negative, and if constipation improves, the score is increasingly positive with a maximum of 5 points, respectively; no change is rated as zero.

**Table 7:** Domains of the final GDQ with corresponding questions.

**GDQ Domain**	**Question number**
Frequency	1
Duration	2, 6
Severity	3, 4, 5
Pain	7, 8, 9, 10
Assistance	11, 12
Consistency	13, 14, 15
Quality of life	16, 17
Development	18

## 4. Discussion

We describe the development and cognitive pretesting and provide clinimetric attributes of the novel self-completed Gut Dysmotility Questionnaire (GDQ) as a quick and comprehensive tool to screen for and monitor gastrointestinal dysmotility of the lower GIT with a focus on constipation in PwPD.

In phase 1, we revealed a lack of symptom-specific (gastrointestinal motility) and disease-specific (PD) validated instruments by a systematic literature review. Instruments such as the NMSQuest (12) and the NMSS (15) that are validated for use in patients with PD, assess several NMS including a domain-entitled gastrointestinal tract with eight and three questions, respectively, asking for dribbling of saliva, dysphagia, and constipation. These instruments aim to assess if there is an involvement of the gastrointestinal tract or not. In contrast, the SCOPA-AUT (16) obtains more detailed information about the whole GIT and autonomic symptoms. In addition, there is the GIDS-PD, which has also been newly developed and validated in PD to assess gastrointestinal dysfunction including the entire GIT (36). However, there is no questionnaire, which focuses on the lower GIT and covers symptoms of dysmotility and constipation.

The second issue we revealed in phase 1 was a wide range of diverse definitions of constipation as also identified in previous studies (37). Therefore, we applied the Rome IV criteria, the gold standard for gastroenterologists, in defining criteria for assessing gastrointestinal disorders as well as for diagnosing constipation (38).

Moreover, the period to be covered by the questionnaire was challenging to define. It should not exceed the recall period but also be unaffected by short-term influencing factors such as the consumption of specific food or infections. The final consensus was 3 months, also taking into account international expert consortia and the Rome IV criteria (38).

Potential questions and associated domains were identified in the literature review, then compiled, and discussed in repetitive national and international expert consortia involving different disciplines. The technique of questioning, the wording, and the type of response options were also discussed. We decided to use four-item response options in the form of multiple-choice answers for all questions, except for the domain consistency and development, for which we used a table and a visual analog scale, respectively.

Phase 1 resulted in the preliminary GDQ. A limitation of phase 1 was that not all critiques could be included in the preliminary questionnaire as these would have been inappropriate for everyday clinical use (e.g., free text answers), would have greatly lengthened the questionnaire (e.g., assessment of co-morbidities and influencing factors on the GIT such as habits and medical therapy), or was believed to have arisen from a feeling of shame about some questions.

The gold standard for developing qualitative questionnaires is cognitive pretests, which we conducted in phase 2 (30). The cognitive pretest of the pGDQ combined quantitative and qualitative methods including interviews and evaluation questionnaires as this has been proven to be useful and effective for a new questionnaire. Based on similar studies on testing questionnaires and referring to cost-benefit considerations in the published literature, an average of 20 people per cognitive pretest is recommended due to the high volume of collected data per individual (30). A statistical case number estimation is not possible when performing cognitive pretests. We included 21 PwPD and 30 age- and sex-matched HC in phase 2a. The cognitive pretests led to changes in the selection of questions, the technique of questioning and their structure, the kind and structure of answer options, as well as the wording. Significantly more and precise criticisms were collected in the oral interviews, especially with the method of thinking aloud, than in the evaluation questionnaires (355 vs. 72). This was accounted by a greater willingness of participants to declare criticisms orally in a conversation than in written form. In addition, in PwPD, writing can be restricted by motor symptoms. This is an important finding and shows the necessity of guided interviews in scale development even though this means a considerably higher time commitment. In our experience, interviews could last more than 3 h, particularly with advanced PwPD. In contrast, the evaluation questionnaires of PwPD and HC provided valuable feedback about the improvements after adjusting the questionnaire to the results of phase 2a.

A major criticism was expressed by PwPD and HC in relation to questions, which included sub-questions and the need to skip questions dependent on the previous answer. Furthermore, including a variety of response options such as multiple-choice, scales, and tables proved to be impractical, error-prone, and demotivating for the participants. In particular, question 12 about stool consistency, which was designed as a table, was split into individual multiple-choice questions to achieve a more continuous method of collection. Question 16 about the development of constipation, which was recorded as a scale, was adapted with clear boxes to tick including numeric values and an additional option to record, i.e., “no constipation.” It was also removed from the overall rating and is designed to stand alone for the evaluation of the development of constipation intended to serve as a progress indicator for the neurologists in addition to the total score. By equalization of the design of questions as well as answer options to a 4-point multiple-choice response, ranging from no symptoms (0 points) to the worst symptoms (3 points) with the zero-point answer always being the first answer option, we could minimize confusion, and it helped to streamline the answering process as confirmed by PwPD and HC in phase 2b. Hereby, the calculation of the total score of the questionnaire improved. In phase 2a, we revealed a relevant number of total scores that were incorrectly calculated, whereas in phase 2b, all total scores were correct. This can also be referred to the scoring guide which was greatly simplified and proved to be quick to learn, easy to implement, and less prone to errors. The streamlining of the questionnaire is also objectively reflected in the required median completion time, which was reduced from 6 to 4 min. In addition, PwPD, HC, and neurologists reported improvements in the evaluation questionnaires of phase 2b in comparison to 2a in relation to relevance, comprehensiveness, length, and comprehensibility of questions and answers of the pfGDQ in comparison to the pGDQ. Sudman and Bradburn (39) said, “Even after years of experience, no expert can write a perfect questionnaire.”

The data quality of phase 2 was very satisfactory with all included participants being fully computable. Reliable responses of the self-completed questionnaires were secured by regular results in the cognitive assessment. The study group of PwPD can be evaluated as a representative group for PD as PwPD throughout all disease stages from newly diagnosed drug-naive PwPD to advanced PwPD with disease durations up to 21 years, and high LEDD were investigated (Tables 1, 4, 6). Furthermore, PwPD showed on average an intermediate motor burden based on the H&Y stage and were evaluated as moderately ill in the CGI-S (Table 1). PwPD presented with more NMS and worse HRQoL in comparison to HC as expected (12, 40). Gastrointestinal dysmotility and constipation were also significantly more common in PwPD than in HC. This was found in the established validated questionnaires and scales as well as in the pGDQ (Tables 1, 2, 4). All pGDQ domains except the domain development showed a high association with the pGDQ total score as well as the pGDQ total score with the NMSQuest total score as a measure of general NMS burden and the SCOPA-AUT total score as a measure of gastrointestinal and autonomic symptoms (Table 3). Furthermore, the pGDQ total score and its domains were tested against corresponding individual questions of these validated instruments (Table 3). We found significant correlations primarily on a moderate and high level. These findings provide good construct validity of the pGDQ. We also used the PDQ-8, a validated measure of HRQoL in PD, as a further measure for convergent validity. The similar content of the pGDQ domains with the independent corresponding measures explains the high correlations but also reflects that these symptoms can be assessed in a simpler and brief way, which is relevant for routine assessments in clinics. Constipation is a known symptom of depression, independent of PD, so that a significant correlation of the pGDQ and the BDI in PwPD and HC on a lower level was expected (41). This was indeed the case with a correlation on a moderate level further supporting the discriminant validity of the pGDQ. Furthermore, the observed strong correlation between memory and constipation has also been discussed in the literature (42).

In the pGDQ and pfGDQ, a high-floor effect was found for some questions and domains. This was expected since not every participant exhibited all the characteristics of gut dysmotility so that this high-floor effect was particularly pronounced in the control group. However, the number of study participants is relatively small for this kind of analysis, so that in the validation study with a larger sample size, it has to be clarified whether these reflect sample characteristics or scale properties. There was no relevant ceiling effect. For a phase 2 study, these findings indicate a suitable acceptability of the questionnaire.

In the clinimetric statistics of the pGDQ questions containing sub-questions, the domains that included these questions (mainly the domain pain) as well as the domain consistency and development with different types of response options stood out negatively. This was supported by the results of the interviews and evaluation questionnaires. Subsequently, main adjustments were performed in relation to these questions and domains.

The pGDQ and the pfGDQ demonstrated excellent internal consistency (Cronbach‘s alpha value up to 0.92 and 0.94).

Limitations of the phase 2 studies were mainly related to the performance of specific analyses such as the evaluation of floor/ceiling effects as discussed above, the evaluation of temporal reliability by a retest, and the definition of cutoff scores to discriminate between participants with and without constipation. This is linked to the small number of participants in cognitive pretest studies in comparison to validation studies. However, the number of participants in this cognitive pretest study was higher for PwPD and HC than recommended (30).

Phase 2 resulted in the final GDQ that enquires in 18 questions with detailed information about gastrointestinal dysmotility with a focus on constipation during the past 3 months and covers eight domains including the effect of bowel movements on HRQoL and the development of constipation (Figure 3; Table 7). The GDQ showed both high acceptance and effectiveness in assessing gastrointestinal dysmotility in PwPD and HC as well as sufficient reliability and construct validity. The self-completed GDQ can be used as a comprehensive, simple, and quick instrument for screening and monitoring gastrointestinal dysmotility in PwPD and HC. Furthermore, the length of time required for completion by the patients as well as evaluation by the physicians is a few minutes so that the GDQ can easily be integrated into clinical practice (Figure 4). How valuable the GDQ is for measuring changes in gastrointestinal dysmotility after treatment or in the course of PD needs to be assessed in further studies. Even though we performed an intensive cognitive pretesting to create the GDQ, an international validation study with a higher number of PwPD and HC including a retest to investigate temporal reliability is planned.

## Data availability statement

The raw data supporting the conclusions of this article will be made available by the authors, without undue reservation.

## Ethics statement

The studies involving human participants were reviewed and approved by Technische Universität Dresden, Dresden, Germany. The patients/participants provided their written informed consent to participate in this study.

## Author contributions

VR and LK conceptualized the project, had a major role in the study execution of phase 1 and 2, analyzed data, and wrote the manuscript. LB analyzed data. KRC, RS, JH, SB, ZK, VL, BF, HR, AR, and RU had a major role in the study execution of phase 1. RU, AF, BF, and HR had a major role in the study execution of phase 2. BF, HR, AR, and AS performed the cross-cultural adaptation. All authors critically revised the manuscript for intellectual content and approved the final draft.
