# Supplementary material for: The Gut Dysmotility Questionnaire for Parkinson's disease: Insights into development and pretest studies
Source: Front Neurol. 2023 Mar 28;14:1149604. doi: 10.3389/fneur.2023.1149604 (PMC10086186; doi:10.3389/fneur.2023.1149604)
Supplement: Supplementary file 1 [file Data_Sheet_1.pdf]

Name: ______________________________ Today's date: ____________

Sex: ☐ male ☐ female ☐ divers Age: _________ years

GUT DYSMOTILITY QUESTIONNAIRE (GDQ)

This questionnaire is asking about your gastrointestinal function during the **past 3 months.** Please answer the questions by marking the boxes. For each question only one answer is allowed. Please mark the answer, which describes your situation within the **past 3 months** the best.

The questionnaire is meant to be self-completed. Please answer the questions the best you can, if needed with support of your carer.

1. How many successful bowel movements per week have you usually had during the past 3 months?

☐ 5 times per week or more ☐ 3 – 4 times per week ☐ Twice per week ☐ Once per week or less

2. Constipation can be described as having less than three bowel movements per week. For how many years have you had less than three bowel movements per week?

☐ Not applicable ☐ Less than 5 years ☐ Between 5 and 10 years ☐ More than 10 years

3. How often did you have to squeeze to pass stool during the past 3 months?

☐ Never ☐ Rarely/sometimes ☐ Often ☐ Mostly/always

4. How often have you felt a constriction or blockage (in your anus), which made it difficult to pass stool during the past 3 months?

☐ Never ☐ Rarely/sometimes ☐ Often ☐ Mostly/always

5. How often have you felt that your bowel movement was incomplete after passing stool during the past 3 months?

☐ Never ☐ Rarely/sometimes ☐ Often ☐ Mostly/always

6. How much time did you need per bowel movement on average during the past 3 months?

☐ Less than 5 minutes ☐ Between 5 and 10 minutes ☐ Between 10 and 20 minutes ☐ More than 20 minutes

7. How often have you felt a painful pull in your stomach or unpleasant bloating during the past 3 months?

☐ Never ☐ Rarely/sometimes ☐ Often ☐ Mostly/always

8. How <u>severe</u> was the painful pull in your stomach or unpleasant bloating during the past 3 months?

☐ Not applicable ☐ Mild ☐ Moderate ☐ Severe

9. How often have you suffered from rectal pain (in your anus) during a bowel movement or from an unpleasant rectal sensation (fullness) during the past 3 months?

☐ Never ☐ Rarely/sometimes ☐ Often ☐ Mostly/always

10. How <u>severe</u> was your rectal pain (in your anus) during a bowel movement or the unpleasant rectal sensation (fullness) during the past 3 months?

☐ Not applicable ☐ Mild ☐ Moderate ☐ Severe

11. How often have you used laxatives (medicine/food) during the past 3 months?

☐ Never ☐ Rarely/sometimes ☐ Often ☐ Mostly/always

12. Have you used enemas or manual help to empty your bowels during the past 3 months?

☐ Never ☐ Rarely/sometimes ☐ Often ☐ Mostly/always

13. How often was the consistency of your stool liquid during the past 3 months?

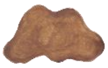

☐ Never ☐ Rarely/sometimes ☐ Often ☐ Mostly/always

14. How often was the consistency of your stool soft during the past 3 months?
(For example like a sausage)

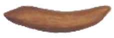

☐ Mostly/always ☐ Often ☐ Rarely/sometimes ☐ Never

15. How often was the consistency of your stool hard during the past 3 months?
(For example like a lumpy sausage or separate lumps)

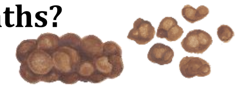

☐ Never ☐ Rarely/sometimes ☐ Often ☐ Mostly/always

16. Have you been satisfied with your bowel activity during the past 3 months?

☐ Satisfied ☐ Somewhat dissatisfied ☐ Moderately dissatisfied ☐ Very dissatisfied

17. Have your bowel movements affected your activities of daily life during the past 3 months? *(For example, due to physical discomfort, the duration of toilet visits, by thoughts about this issue.)*

☐ No, none or minor difficulties. My activities or my well-being are not affected.
☐ Yes, some difficulties, which affect my activities or my well-being.
☐ Yes, severe difficulties, which affect my activities or my well-being, but do not stop me from any activities.
☐ Yes, very severe difficulties, which affect my activities or my well-being and stop me from activities.

18. Please mark the box, which best describes the development of your constipation during the past 3 months:

☐ No constipation

OR

☐	☐	☐	☐	☐	☐	☐	☐	☐	☐	☐
-5	-4	-3	-2	-1	0	1	2	3	4	5
Getting worse					No change					Getting better

Thank you for completing this questionnaire.
May we ask who completed this questionnaire?

☐ Patient ☐ Carer ☐ Patient and carer
